# Supplementary material for: A new Majorana platform in an Fe-As bilayer superconductor
Source: Nat Commun. 2020 Nov 10;11:5688. doi: 10.1038/s41467-020-19487-1 (PMC7655862; doi:10.1038/s41467-020-19487-1)
Supplement: Supplementary file 1 — Supplementary Information [file 41467_2020_19487_MOESM1_ESM.pdf]

Supplementary Information for:

A new Majorana platform in an Fe-As bilayer superconductor

Wenyao Liu[1,2,9], Lu Cao[1,2,9], Shiyu Zhu[1,2,9], Lingyuan Kong[1,2,9], Guangwei Wang[3], Michał Papaj[4], Peng Zhang[5], Ya-Bin Liu[6], Hui Chen[1,2], Geng Li[1,2], Fazhi Yang[1,2], Takeshi Kondo[5], Shixuan Du[1,7], Guang-Han Cao[6], Shik Shin[5], Liang Fu[4], Zhiping Yin[3], Hong-Jun Gao[1,2,7*] and Hong Ding[1,2,7,8*]

[1]Beijing National Laboratory for Condensed Matter Physics and Institute of Physics, Chinese Academy of Sciences, Beijing 100190, China

[2]School of Physical Sciences, University of Chinese Academy of Sciences, Beijing 100190, China

[3]Department of Physics and Center for Advanced Quantum Studies, Beijing Normal University, Beijing 100875, China

[4]Department of Physics, Massachusetts Institute of Technology, Cambridge, Massachusetts 02139, USA

[5]Institute for Solid State Physics, University of Tokyo, Kashiwa, Chiba 277-8581, Japan

[6]Department of Physics, Zhejiang University, Hangzhou 310027, P. R. China

[7]CAS Center for Excellence in Topological Quantum Computation, University of Chinese Academy of Sciences, Beijing 100190, China

[8]Songshan Lake Materials Laboratory, Dongguan, Guangdong 523808, China

[9]These authors contributed equally: Wenyao Liu, Lu Cao, Shiyu Zhu, Lingyuan Kong

[*]E-mail: hjgao@iphy.ac.cn; dingh@iphy.ac.cn

SUPPLEMENTARY NOTE 1: Details of DFT + DMFT band calculations

To take into account the electronic correlation effect established in iron arsenide superconductors, a combination of density functional theory and dynamical mean field theory (DFT + DMFT) was used to compute the electronic structure and topological properties of $CaKFe_4As_4$ in the paramagnetic state [1]. The density functional theory (DFT) part is based on the full-potential linear augmented plane wave method implemented in Wien2K in conjunction with Perdew–Burke–Ernzerhof generalized gradient approximation of the exchange correlation functional [2,3]. DFT + DMFT was implemented on top of Wien2K [4]. In the DFT + DMFT calculations, the electronic charge was computed self-consistently on DFT + DMFT density matrix. The quantum impurity problem was solved by the continuous time quantum Monte Carlo (CTQMC) method with a Hubbard U = 5.0 eV and Hund's rule coupling J = 0.8 eV in the paramagnetic state [5-9]. The experimental crystal structure (space group P4/mmm, #129) of $CaKFe_4As_4$ with lattice constants $a = b = 3.866$ Å and $c = 12.817$ Å was used in the calculations, whereas the As position was optimized by minimizing the force acting on the As atoms in the DFT + DMFT calculations [10-12].

After full charge self-consistency of the DFT + DMFT calculations, we obtained the band structure of $CaKFe_4As_4$ and parameterized the band structure around the Fermi level in terms of tight-binding hopping parameters of Fe 3*d* and As 4*p* orbitals (supplementary figure 1a-b). DFT tight-binding hopping parameters were used as a starting point, and were obtained using the maximally-localized Wannier functions (MLWF) method implemented in the WANNIER90 code [13,14]. The band structure obtained from the MLWF tight-binding model fits well to the first-principles calculated band structures in the range of -5.5 to 2.7 eV relative to the Fermi energy. The DFT tight-binding hopping parameters were renormalized to reproduce the DFT + DMFT band structure in order to obtain the DFT + DMFT tight-binding Hamiltonian. The surface states were calculated through the iterative Green's function method implemented in the WANNIERTOOLS package using the DFT + DMFT tight-binding Hamiltonian [15].

SUPPLEMENTARY NOTE 2: Detail of multi-Gaussian-peak fit for vortex core states

As mentioned in the main text, we applied the multi-Gaussian-peak fit for all *dI*/*dV* spectra inside a vortex to extract the accurate locations of each bound state. Supplementary figure 2 displays the fitting result for several spectra from Fig. 3g.

Note that the vortex shown in supplementary figure 2a is the same as the one in Fig. 3e of the main text.

When the magnetic field exceeds H_{c1} of type-II superconductors, electrons are no longer paired inside a vortex, instead they reform CdGM states at low energies and quasi continuous states at high energies [16]. Therefore, we used a broad Gaussian curve to fit the continuous background states at the high energies, and multiple sharp Gaussian peaks to fit the sharp modes inside the vortex. Supplementary figure 2b shows the STS#1 measured at the vortex center, where the raw data (red circles), the fitting curve without the background (orange circles), the fitting result (black solid line), the broad background (black dash line) and multiple Gaussian fitting peaks (colored dotted lines) are plotted together. Supplementary figure 2c and supplementary figure 2d show fitting results on the STS#2 and STS#3 whose positions are away from the vortex center and marked in supplementary figure 2a.

SUPPLEMENTARY NOTE 3: Reproducibility of MZM and quantized CBSs

To check the reproducibility of MZM and quantized CBSs in this material, we measured many vortex cores at the same or different regions under different magnetic fields. Here we plot some typical examples of quantized vortex states. In supplementary figure 3, we append the zero-bias conductance maps (the first row), *dI*/*dV* line-cuts plots (the second row) and waterfall like plots (the third row) of vortex#2 to vortex#6. Except vortex#2, which was very close to the vortex#1 (shown in the main text), other vortices are all located at different regions. In addition, vortex#2 to vortex#5 were measured at 2 T and vortex#6 at 4 T. As mentioned, the sizes of vortices#2 to vortex#5 are similar (~ 6.5 nm), while the size of vortex#6 is 5.8 nm due to the higher magnetic field effect. Vortex#2 is very similar to vortex#1 in terms of the level spacing (ΔE ~ 1.22 meV) and the spatial profile of each bound state. In addition, we performed twelve measurements on the region shown in Fig. 3a, all results indicating a high reproducibility of MZM and quantized CBSs in this region. Other regions also displayed the universality of vortices with integer-quantized bound states. Using the same analysis method shown in supplementary note 2 on *dI*/*dV* spectra, the level spacings ΔE = 1.22 meV, 1.02 meV, 1.13 meV, 1.26 meV and 1.15 meV were observed for vortex#2 to vortex#6, respectively.

SUPPLEMENTARY NOTE 4: Numerical simulations of vortex bound states

The procedure used to calculate energies and wavefunctions of the vortex core states largely follows our previous work[2]. For the sake of completeness, we recapitulate the calculation scheme below. We perform calculations based on Bogoliubov-de Gennes (BdG) Hamiltonian for 2D surface states with linear (Dirac) dispersion. In the course of our analysis we found that such a simple model is enough to qualitatively reproduce the features observed in the experiment, even though in general the behavior of iron-based superconductors features a rich variety of phenomena. To fully take into account the effects of disorder, neighboring vortices and multiple bands at the Fermi energy, a large-scale simulation would be required. In principle, superconductivity at each pocket of the Fermi surface contributes their own set of vortex core states. However, one conceivable reason for the surprising simplicity of the spectrum may be the fact that STM is most sensitive to the states around the Γ pocket, where the Dirac surface states exist.

Having acknowledged that, we assume that a vortex with vorticity $|n| = 1$ is placed at the origin, which corresponds to the spatial dependence of the superconducting order parameter given by $\Delta(r,\phi) = \Delta_0 \tanh\frac{r}{\xi}\, e^{i\phi}$, with Δ_0 being the magnitude of the gap and ξ the superconducting coherence length. For the Dirac surface states, the BdG Hamiltonian is:

$$H_{Dirac} = v_F \tau_z (p_x \sigma_x + p_y \sigma_y) - \mu \tau_z + \Delta(r,\phi)\tau_x$$

τ and σ are Pauli matrices describing the particle-hole and spin spaces, respectively, v_F is the Fermi velocity and μ is the chemical potential. We assume rotational symmetry of the vortex, so we can express the BdG equations as a set of 1D radial equations separated into angular momentum modes. We therefore use the following ansatz:

$$\psi(r,\phi) = \frac{e^{i\nu\phi - \frac{i\phi}{2}\sigma_z + i\frac{\pi}{4}\sigma_z + \frac{i\phi}{2}\tau_z}}{\sqrt{r}} \begin{pmatrix} u_\uparrow(r) \\ u_\downarrow(r) \\ v_\downarrow(r) \\ v_\uparrow(r) \end{pmatrix}$$

where ν is the angular momentum. We can express all the lengths in terms of the coherence length $\xi = \frac{\hbar v_F}{\Delta_0}$ and all the energies in terms of Δ_0 to finally obtain the set

of differential equations:

$$\begin{pmatrix} -\bar{\mu} & -\frac{d}{d\bar{r}}-\frac{\nu+\frac{1}{2}}{\bar{r}} & f(\bar{r}) & 0 \\ \frac{d}{d\bar{r}}-\frac{\nu+\frac{1}{2}}{\bar{r}} & -\bar{\mu} & 0 & f(\bar{r}) \\ f(\bar{r}) & 0 & \bar{\mu} & \frac{d}{d\bar{r}}+\frac{\nu-\frac{1}{2}}{\bar{r}} \\ 0 & f(\bar{r}) & -\frac{d}{d\bar{r}}+\frac{\nu-\frac{1}{2}}{\bar{r}} & \bar{\mu} \end{pmatrix} \begin{pmatrix} u_\uparrow(\bar{r}) \\ u_\downarrow(\bar{r}) \\ v_\downarrow(\bar{r}) \\ v_\uparrow(\bar{r}) \end{pmatrix} = E \begin{pmatrix} u_\uparrow(\bar{r}) \\ u_\downarrow(\bar{r}) \\ v_\downarrow(\bar{r}) \\ v_\uparrow(\bar{r}) \end{pmatrix}$$

with $\bar{\mu} = \mu/\Delta_0$ and $\bar{r} = \frac{r}{\xi}$. This set of equations is then discretized on a 1D lattice (equivalent to solving the equations on a disk with radius $R = 100\,\xi$) and lowest lying eigenvalues and eigenvectors are obtained. To avoid the fermion doubling problem, we use the approach of Susskind adjusted to quasi-1D radial geometry [17-19]. The eigenvectors are then used to calculate the local density of states by using:

$$LDOS(E,r) = \frac{1}{r} \sum_{n,\sigma=\uparrow,\downarrow} \left|u_{n,\sigma}(r)\right|^2 \delta(E - E_n) + \left|v_{n,\sigma}(r)\right|^2 \delta(E + E_n)$$

where the sums are taken over the positive eigenvalues and the spin components. The 2D density maps are then obtained from the radial dependence by using the rotational symmetry of the wave functions in our model.

The only fitting parameter used in the calculation described above is the reduced chemical potential $\bar{\mu}$ (expressed in the units of Δ_0, which is taken from the measurements). While introducing a more complicated vortex core radial profile would increase the quantitative agreement with the experiment, it wouldn't qualitatively change the obtained eigenvalues and the local density of states [20], which already compare satisfactorily with the data.

SUPPLEMENTARY NOTE 5: Additional results of spatial patterns in the topological and ordinary vortex cores

In the main text, we display the full consistence of wave functions between the numerical calculation and experimental results for MZM and vortex bound states in

negative energies. Here we plot more results of vortex bound states at both negative and positive energies in supplementary figure 4 and compare them with the calculation results.

Supplementary figure 4a shows the configuration of our experiments. We show the spatial pattern for the first four vortex bound states of vortex#3 (supplementary figure 4b). As we mention in the main text, in the topological vortex, the energies of vortex bound state are approximately $E_n = n\Delta^2/E_F$ (n = 0, ±1, ±2, ±3), n are integers and proportional to angular momentum eigenvalues, the n-th level of vortex bound state is marked by L_n. We notice that only the MZM (L_0) and the first-level of CBS at negative energy (L_{-1}) show the solid-circle pattern, where the first-level of CBS at positive energy (L_{+1}) and other higher energy CBSs ($L_{\pm2}$ & $L_{\pm3}$) show hollow-ring patterns. Remarkably, such a stark asymmetry between the states at L_{-1} and L_{+1} can be well reproduced by a theoretical analysis based on the sign of Dirac point energy.

As we demonstrated above, the wavefunctions of CBS are correlated with the sign of the Dirac point energy. Supplementary figure 4c shows that in the case of our experiment the Dirac point is above the E_F. Our numerical calculation with the parameters (Δ = 5.8 meV, E_F = 20.9 meV, ξ_0 = 6.4 nm) is displayed in supplementary figure 4d. The excellent consistency between experimental data and numerical calculation is obtained once again.

Next, we relocate the Dirac point to below the E_F in our model simulation, while the absolute values of parameters are unchanged (shown in supplementary figure 4e). The numerical calculation result displayed in supplementary figure 4f. Comparing the supplementary figure 4d and supplementary figure 4f, the spatial profiles for L_{-1} and L_{+1} are obviously reversed, where L_{-1} possesses the hollow-ring pattern and L_{+1} possesses the solid-circle pattern.

Finally, we simulated the spatial wavefunction of the ordinary vortex bound states derived from a trivial band (as shown in supplementary figures 4g-j) as well. One can easily see the differences between the calculation of the ordinary vortex and our experimental results. Note that the angular momentum eigenvalues of the ordinary vortex bound states can only be half-odd-integer (causing approximately $E_n = n\Delta^2/E_F$ while n = $\pm\frac{1}{2}, \pm\frac{3}{2}, \pm\frac{5}{2}$ are half-odd-integers), and no zero mode can be worked out. Besides, only single pattern is solid-circle shape in the ordinary vortex while two solid-circle patterns can be observed in the topological vortex.

SUPPLEMENTARY NOTE 6: STM energy resolution at T_{sample} = 0.45 K and bias voltage offset

As reported in previous studies [21, 22], we apply a standard procedure to calibrate the zero-voltage bias of system as shown in supplementary figure 5a. Since the *I-V* curves always intersect the point where the current and bias are both zero, the overlap of *I-V* curves generally gives the offset of the real zero-voltage-bias point. As supplementary figure 5a shown, the system has already been calibrated to the real zero-voltage bias. And all dI/dV spectra and maps are measured after the zero-voltage-bias calibration. We confirm the energy resolution by measuring the dI/dV spectrum of Nb (110) single crystal at T_{exp} = 0.45 K (as supplementary figure 5b shown). Two different methods are used in the calibration. The first one is the same as the previous study. We calculate the d^2I/d^2V numerically from the experimental dI/dV spectrum, which yields a broadening of the superconducting spectrum edge about 0.26 meV. The second one is to fit the tunneling spectrum by the Dynes formula as following:

$$\frac{\mathrm{dI}}{\mathrm{dV}} \propto \int N(E)\frac{df(E+eV)}{dV}dE$$

where

$$\mathrm{N(E)} = \mathrm{Re}\left(\frac{E-i\Gamma}{\sqrt{(E-i\Gamma)^2-\Delta^2}}\right)$$

and the $f(E+eV)$ is the Fermi-Dirac distribution at the effective electron temperature T_{eff}. The fitting gives the Δ = 1.53 meV, Γ = 0.04 meV, and the T_{eff} = 0.69 K. The energy resolution is defined by 3.5 $k_B T_{eff}$ = 0.21 meV. All in all, the energy resolution of our STM system is better than 0.3 meV.

SUPPLEMENTARY NOTE 7: Information about $CaKFe_4As_4$ single crystals

Supplementary figure 6a displays the x-ray diffraction data on (00l) peaks and can be indexed by tetragonal structure (space group P4 / mmm). Supplementary figure 6b exhibits the temperature dependence of the resistivity for the $CaKFe_4As_4$ single crystal, where the superconducting transition temperature T_c of the samples is around 35 K. The narrow temperature-transition section (~ 0.5 K) indicates the superconductivity of the sample is quite homogenous. The Supplementary figure 6c shows the temperature dependence of magnetic susceptibility under magnetic field of 10 Oe for $CaKFe_4As_4$ single crystals. The “new” and “normal” indicate two different growth batches of

$CaKFe_4As_4$ single crystals. The magnetic shielding signals of two batches are corresponding with T_c ~ 35 K. The magnetic shielding fraction is higher than 100%, possibly due to the following reasons: (1) The real field might be a little different from 10 Oe due to the residual field in the superconducting magnet. (2) The sample's orientation is not strictly along H // ab.

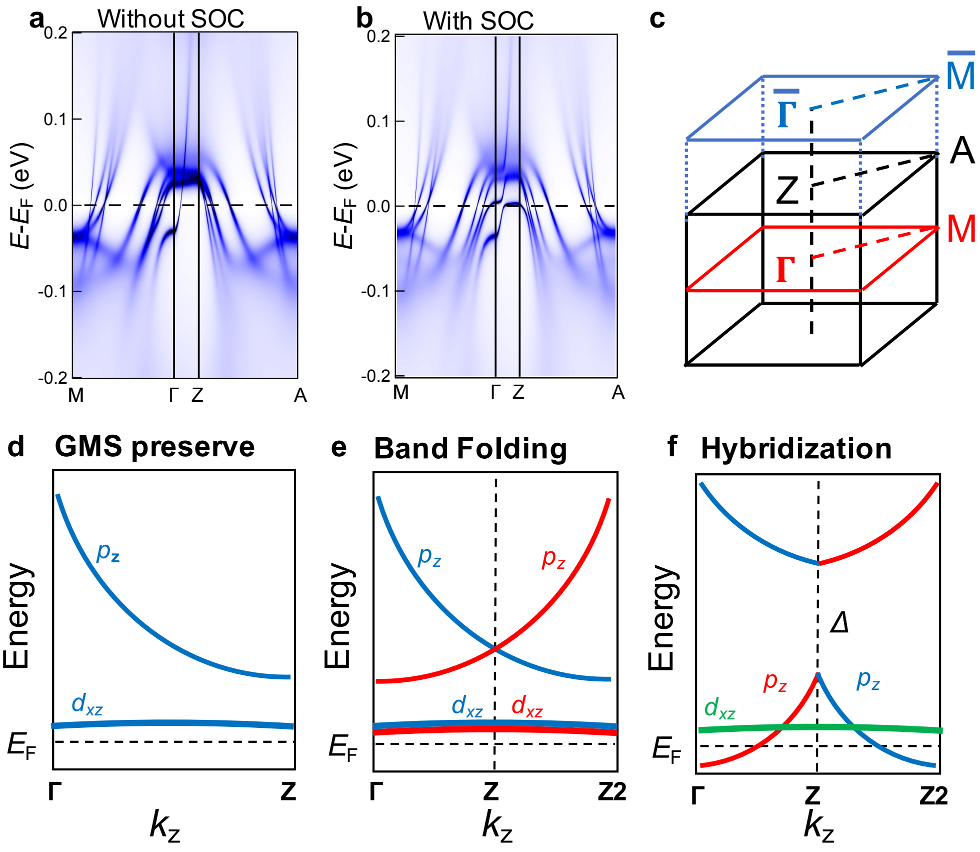

Supplementary Figure 1: **DFT + DMFT results, three-dimensional Brillouin zone and the schematic of the band folding on $CaKFe_4As_4$. a**, Band structure along M-Γ-Z-A without the consideration of spin-orbital coupling. **b**, Band structure with SOC effect. **c**, The projected Brillouin zone of $CaKFe_4As_4$ marks positions of Γ, M, Z and A point. **d,** The original band dispersion (colored as blue) along k_z direction in reciprocal space with glide-mirror symmetry (GMS) preserving. There is no band inversion between p_z and d_{xz} bands. Here we simplify the complicated band structure of $CaKFe_4As_4$ by focusing on the p_z and d_{xz} bands which inducing the topological band inversion. **e,** the folding band (colored as red) after GMS breaking without hybridization at the edge of Brillouin zone (BZ). The original Z point is marked as "Z2" while the new Z point of the folding BZ is marked as "Z" in this figure. **f,** the folding band structure with the hybridization at the edge of the folding BZ. A large hybridized gap pushes the p_z band below the hybridized d_{xz} band (colored as green) causing the topological band inversion.

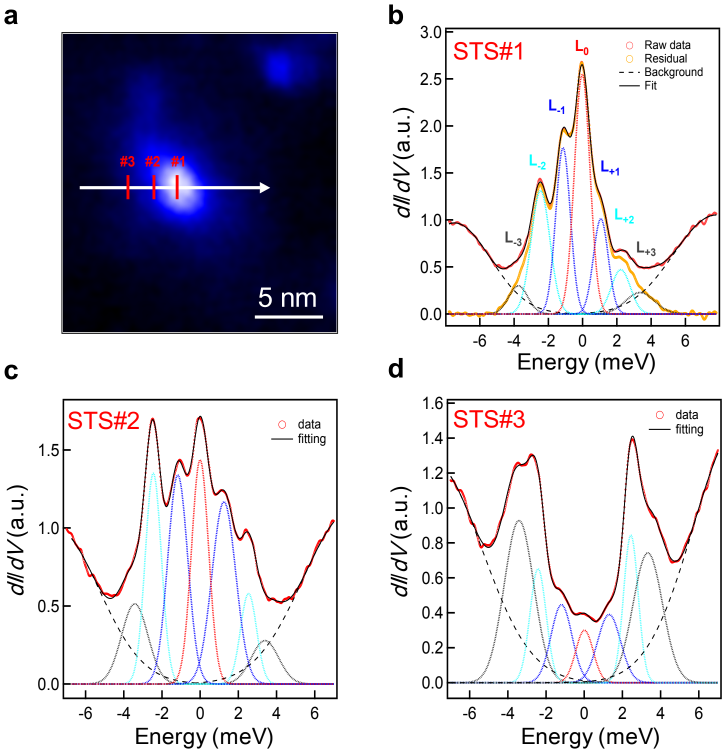

Supplementary Figure 2: Multi-Gaussian-peak fit for *dI*/*dV* spectra in vortex#1. **a**, Zero-bias conductance maps same as Fig. 3d for vortex#1, **b-d**, *dI*/*dV* spectra at different locations marked by red lines in **(a)**. **b**, raw data (red circles), data with the background (orange circles), the background (black dash line), multiple Gaussian fitting peaks (colored dotted lines), and the total fitting curve (black solid line) for STS#1, which is at the vortex center. **c-d**, fitting result using the same fitting procedure for STS#2 and STS#3, which are away from the vortex center.

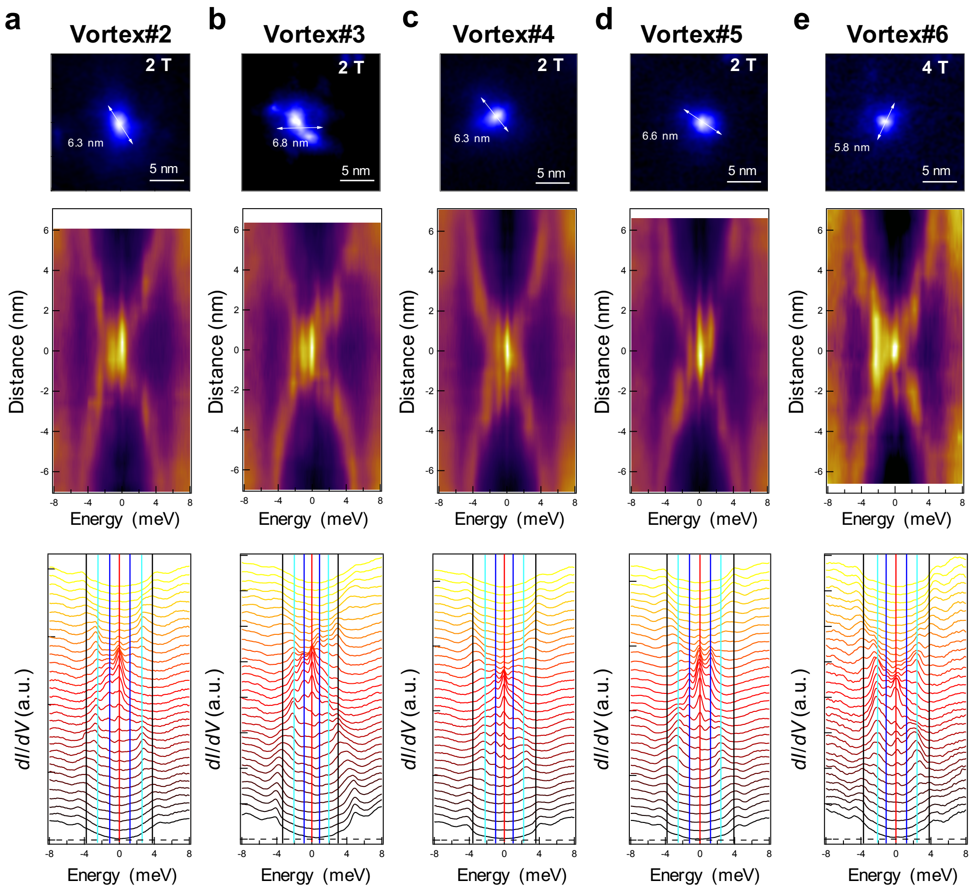

Supplementary Figure 3: **Examples of integer-quantized vortex states at different regions and magnetic fields. a-e**, Zero-bias conductance maps, intensity line-cut plots, and waterfall-like plots of vortex bound states are displayed in the first, second and third row, respectively.

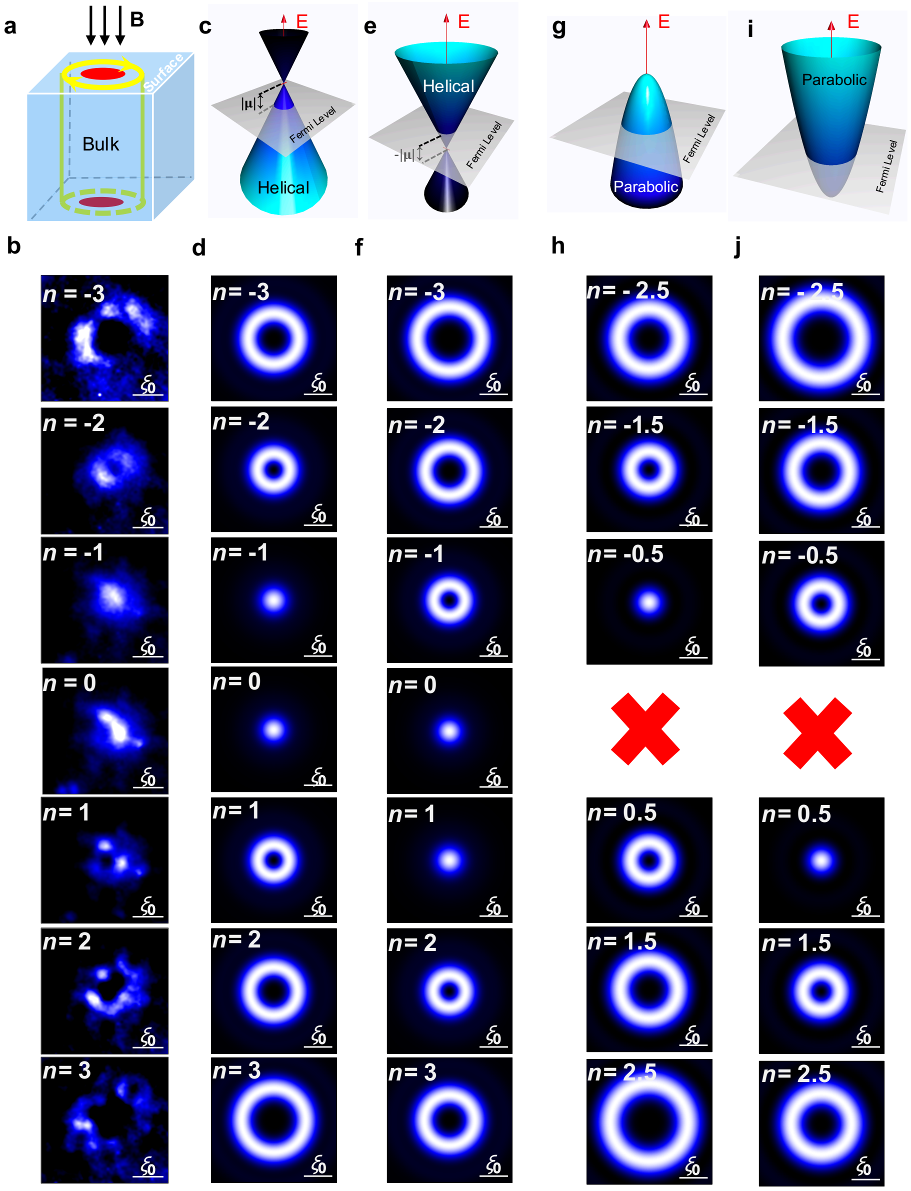

Supplementary Figure 4: **Additional experimental and numerical results for spatial pattern of vortex bound states. a**, Magnetic field configuration in experiment. **b**, Spatial patterns of the vortex bound states with L_{-3}, L_{-2}, L_{-1}, L_0, L_{+1}, L_{+2}, L_{+3} respectively at vortex#3. The size of the area is scaled by the coherence length ξ_0. **c**, A Dirac surface state with its Dirac point above the Fermi level. **d**, The corresponding numerical calculations for (c), the two-dimensional local density of states of each level are shown in order. **e-f**, Same as (c-d) but for the Dirac point below the Fermi level. **g,** A hole-like parabolic band to simulate the trivial band case. **h**, The corresponding numerical two-dimensional local density of states for (g) are shown in order. Note that in the trivial vortex, the eigenvalues of angular momentums are half-odd-integer, and there is no state at zero energy, same as (g-h) but for the electron-like band.

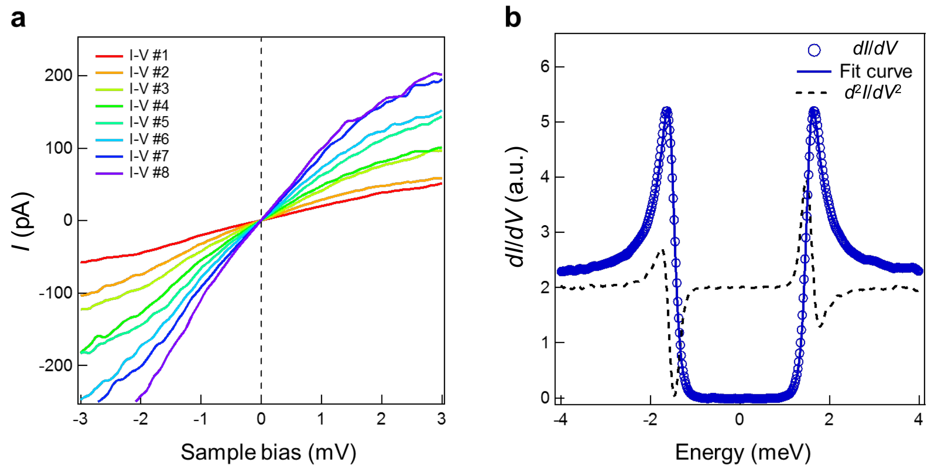

Supplementary Figure 5: Voltage offset and energy resolution calibrations. a, 8 *I*-*V* curves with different values of tunneling resistance overlap at one point, where the true zero current should have a true zero voltage, indicating the exact zero bias of system. All *dI*/*dV* curves are obtained from lock-in techniques simultaneously. b, Superconducting gap of a Nb single crystal measured at 0.45 K / 0 T. The total broadening of the superconducting gap edge is 0.26 meV.

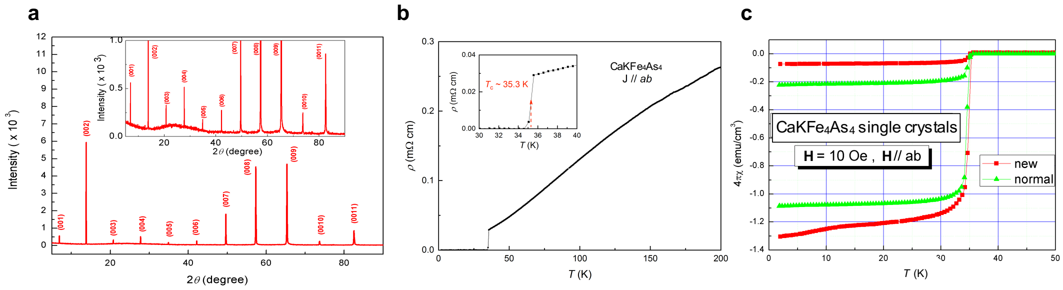

Supplementary Figure 6: a, X-ray diffraction pattern of $CaKFe_4As_4$ single crystal at room temperature. **b,** Temperature dependence of the resistivity ρ for $CaKFe_4As_4$ single crystal. The inset exhibits enlargement near the superconducting transition at low temperature. **c,** Temperature dependence of magnetic susceptibility under magnetic field of 10 Oe for $CaKFe_4As_4$ single crystals. “new” indicates that $CaKFe_4As_4$ grown recently and “normal” indicates previous high quality $CaKFe_4As_4$ single crystals.

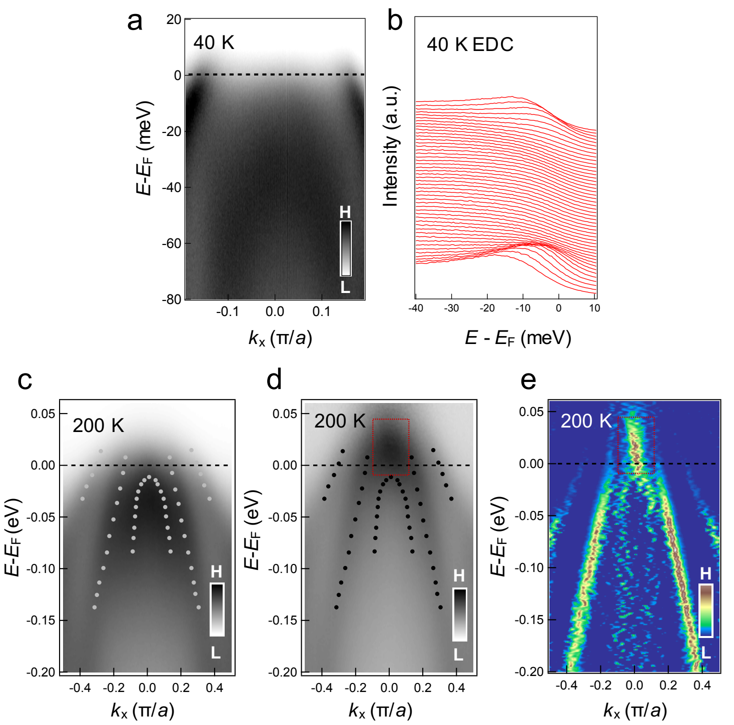

Supplementary Figure 7: **a**, A laser-based-ARPES spectral intensity plot along Γ-M, measured with a *p*-polarized 7-eV laser at 40 K. **b**, EDCs at 40 K. **c**, The band structure measured at the photon energy of 34 eV along Γ-M at the temperature of 200 K. The gray dots guide the band dispersion and are same in (**d**) as the black dots. **d**, The ARPES intensity plot from (**c**) with each energy distribution curve (EDC) dividing the 200-K Fermi-Dirac function. The red box indicates the same area in (**e**). **e**, The MDC curvature plot of the data in (**c**) [23]. The area in the red box displays a band crossing.

SUPPLEMENTARY REFERENCES

[1] Kotliar, G. et al. Electronic structure calculations with dynamical mean-field theory. *Rev. Mod. Phys.* **78**, 865 (2006).

[2] Blaha, P. et al. *WIEN2K, AN AUGMENTED PLANE WAVE + LOCAL ORBITALS PROGRAM for CALCULATING CRYSTAL PROPERTIES*. Karlheinz Schwarz, Techn. Universität Wien, Austria (2001).

[3] Perdew, J. P. et al. Generalized gradient approximation made simple. *Phys. Rev. Lett.* **77**, 3865-3868 (1996).

[4] Haule, K. et al. Dynamical mean-field theory within the full-potential methods: Electronic structure of $CeIrIn_5$, $CeCoIn_5$, and $CeRhIn_5$. *Phys. Rev. B* **81**, 195107 (2010).

[5] Haule, K. Quantum onte Carlo impurity solver for cluster dynamical mean-field theory and electronic structure calculations with adjustable cluster base. *Phys. Rev. B* **75**, 155113 (2007).

[6] Werner, P. et al. Continuous-time solver for quantum impurity models. *Phys. Rev. Lett.* **97**, 076405 (2006).

[7] Yin, Z. P. et al. Kinetic frustration and the nature of the magnetic and paramagnetic states in iron pnictides and iron chalcogenides. *Nat. Phys.* **7**, 294 (2011).

[8] Yin, Z. P. et al. Kinetic frustration and the nature of the magnetic and paramagnetic states in iron pnictides and iron chalcogenides. *Nat. Mater.* **10**, 932 (2011).

[9] Yin, Z. P. et al. Spin dynamics and orbital-antiphase pairing symmetry in iron-based superconductors. *Nat. Phys.* **10**, 845 (2014).

[10] Akira, I. et al. New-structure-type Fe-based superconductors: $CaAFe_4As_4$ (A = K, Rb, Cs) and $SrAFe_4As_4$ (A = Rb, Cs). *J. Am. Chem. Soc.* **138,** 3410-3415 (2016).

[11] Haule, K. & Birol, T. Free energy from stationary implementation of the DFT + DMFT functional. *Phys. Rev. Lett.* **115**, 256402 (2015).

[12] Haule, K. & Pascut, G. L. Forces for structural optimizations in correlated materials within a DFT +embedded DMFT functional approach. *Phys. Rev. B* **94**, 195146 (2016).

[13] Marzari, N. et al. Maximally localized Wannier functions: theory and applications. *Rev. Mod. Phys.* **84**, 1419 (2012).

[14] Mostofi, A. A. et al. An updated version of wannier90: a tool for obtaining maximally-localized Wannier functions. *Comput. Phys. Commun.* **185**, 2309 (2014).

[15] Wu, Q. S. et al. Wannier Tools: An open-source software package for novel topological materials. *Comput. Phys. Commun.* **224**, 405 (2018).

[16] Suderow, H. et al. Imaging superconducting vortex cores and lattices with a scanning tunneling microscope. *Supercond. Sci. Technol.* **27**, 063001 (2014).

[17] Susskind, L. Lattice fermions. *Phys. Rev. D* **16**, 3031 (1977).

[18] Stacey, R. Eliminating lattice fermion doubling. *Phys. Rev. D* **26**, 468 (1982).

[19] Gutiérrez, C. et al. Interaction-driven quantum Hall wedding cake–like structures in graphene quantum dots. *Science* **361**, 789–794 (2018).

[20] Berthod, C. et al. Vorticity and vortex-core states in type-II superconductors. *Phys. Rev. B* **71**, 134513 (2005).

[21] Wang, D. et al. Evidence for Majorana bound states in an iron-based superconductor. *Science* **362**, 333–335 (2018).

[22] Kong, L. et al. Half-integer level shift of vortex bound states in an iron-based superconductor. *Nat. Phys.* **15**, 1181–1187 (2019).

[23] Zhang, P. et al. A precise method for visualizing dispersive features in image plots. *Rev. Sci. Instrum.* **82**, 043712 (2011).
